# Osteosarcopenic Obesity Syndrome: What Is It and How Can It Be Identified and Diagnosed?

**DOI:** 10.1155/2016/7325973

**Published:** 2016-09-07

**Authors:** Jasminka Z. Ilich, Owen J. Kelly, Julia E. Inglis

**Affiliations:** ^1^Department of Nutrition, Food and Exercise Sciences, Florida State University, Tallahassee, FL, USA; ^2^Abbott Nutrition, Columbus, OH, USA

## Abstract

Conditions related to body composition and aging, such as osteopenic obesity, sarcopenia/sarcopenic obesity, and the newly termed osteosarcopenic obesity (triad of bone muscle and adipose tissue impairment), are beginning to gain recognition. However there is still a* lack of definitive diagnostic criteria* for these conditions. Little is known about the long-term impact of these combined conditions of osteoporosis, sarcopenia, and obesity in older adults. Many may go undiagnosed and progress untreated. Therefore, the objective of this research is to create* diagnostic criteria* for osteosarcopenic obesity in older women. The proposed* diagnostic criteria *are based on* two types of assessments: physical,* via body composition measurements, and* functional,* via physical performance measures. Body composition measurements such as *T*-scores for bone mineral density, appendicular lean mass for sarcopenia, and percent body fat could all be obtained via dual energy X-ray absorptiometry. Physical performance tests: handgrip strength, one-leg stance, walking speed, and sit-to-stand could be assessed with minimal equipment. A score could then be obtained to measure functional decline in the older adult. For* diagnosing osteosarcopenic obesity* and other conditions related to bone loss and muscle loss combined with obesity, a combination of measures may more adequately improve the assessment process.

## 1. Osteosarcopenic Obesity Syndrome: The Triad of Bone, Muscle, and Adipose Tissue Impairment

We recently outlined a new syndrome and termed it* osteosarcopenic obesity* (OSO), signifying the impairment of bone, muscle, and adipose tissues as an ultimate consequence of aging [1]. We also outlined possible nutritional causes and subsequent nutritional prevention and treatments for the OSO syndrome [2, 3]. OSO may also develop due to the initiating presence of overweight/obesity perpetuated by low-grade chronic inflammation, as well as due to inadequate diet and lifestyle [1, 4, 5]. Additionally, some chronic conditions, like cancers, diabetes, and other diseases that cause endocrine imbalance and stem cell lineage disruption, leading to impairment in body composition, may also cause OSO [1, 6]. Although the tight connection between bone and muscle has been recognized and addressed in recent years [7–9], the inclusion of fat tissue, either as an overt obesity, as an age-induced redistribution of fat, or as an infiltrated fat into bone and muscle, is just beginning to gain more attention within the context of bone and muscle impairments [1].

**Figure 1:**
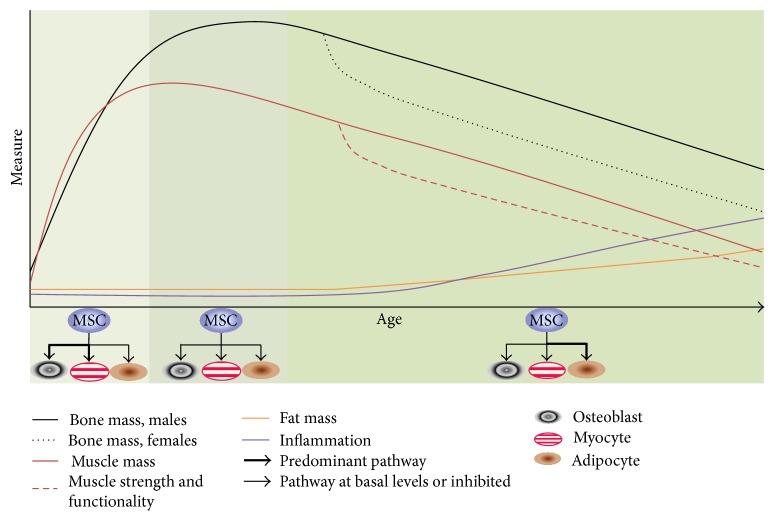
Age-related changes in bone, muscle, and fat tissues and related factors.

Therefore, in our proof-of-the-concept paper [1], we also introduced a new term,* osteopenic obesity*, a previously unrecognized impairment, unrecognized probably because, for years, obesity was considered to be protective for bones. We discussed the importance of changes with aging in bone relative to sarcopenia and adiposity and in view of the critical role of bone in locomotion and thus functionality. Bone and muscle mass/strength decline with age, while body fat increases. These changes in body composition are accompanied by increased low-grade chronic inflammation and a decline in physical activity, a combination that favors OSO [1, 5] (Figure 1). In addition to the physical changes in bone, muscle, and fat, anabolic hormones decline with age. The decline in growth hormone, often referred to as somatopause [10], is well documented. There is also a decline in insulin-like growth factor-1 (IGF-1) [11] associated with aging, but this may also be linked to the decline in growth hormone. In addition, an age-associated decline in estrogen and testosterone is well recognized in both men [12] and women [13]. Figure 1 summarizes this concept: as we age, inflammation increases, leading to shift in mesenchymal stem cell (MSC) lineage commitment that favors greater adipogenesis in bone and muscle, as well as in fat tissues [1]. Ultimately, this deregulation of MSC lineage commitmnt may contribute to many chronic diseases, including osteoporosis and obesity and subsequent decline in functionality [4, 5, 14].

We also highlighted the cellular connections between bone, muscle, and fat and put forward potential cellular mechanisms for development and progression of OSO, as well as changes in bone, muscle, and fat cross talk via alterations in* osteokine*, myokine, and adipokine concentrations, respectively [1]. There are three main phases of stem cell lineage commitment in this model: (1) growth, where osteoblasts and myocytes dominate, muscle and bone are built, and adipogenesis is at basal levels, (2) maintenance, where all three cell types are maintained through remodeling and tissue recovery/repair and adipogenesis is still essentially at basal levels, and (3) deregulation, where adipogenesis predominates while osteoblastogenesis and myogenesis are reduced followed by a reduction in bone and muscle mass and strength and subsequent fat infiltration into those two tissues. Increased low-grade chronic inflammation and aging* per se* are central to this phase (Figure 1).

**Figure 2:**
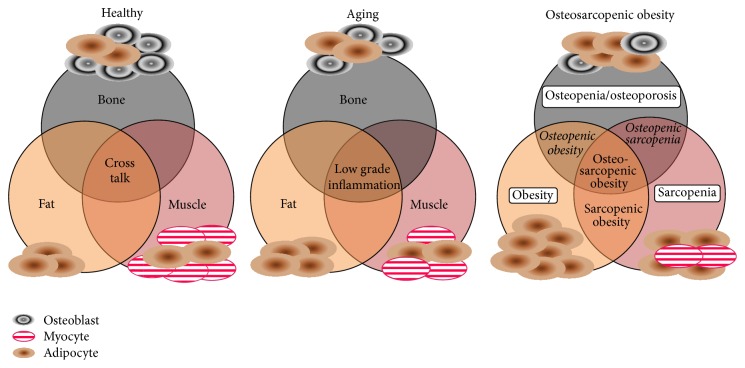
Conceptual model of bone, muscle, and fat tissues in healthy and diseased states: osteosarcopenic obesity is the most advanced stage resulting from aging or other compromised impairment in bone, muscle, and adipose tissue.

As the stem cells age, or as their normal regulatory processes are modulated by adiposity and/or low-grade chronic inflammation, the infiltration of fat cells into muscle and bone becomes evident and replacement of muscle and bone cells by fat cells ensues (Figure 2). In our model, OSO is considered the most advanced impaired stage of bone, muscle, and fat tissues. The other possible conditions in this model include* osteopenic obesity* and* osteopenic sarcopenia*, in addition to the already recognized sarcopenia and sarcopenic obesity. Each arises depending on where the fat infiltration is predominant, or perhaps where the fat accumulation originated. Thus, all conditions might eventually result in osteosarcopenic obesity, with time. For example, sarcopenic obesity would be the result of increased fat mass and fat infiltration into muscle (the causes can be multifactorial and would include diet and lifestyle but would depend on the individual), leading to lower muscle mass, quality, and functionality and possibly increased frailty. Decreased locomotion, reduced muscle mass, and improper nutrition, in combination with age and/or inflammation, would either induce or accelerate the fat infiltration into bone leading to osteopenic obesity and when compounded with sarcopenia eventual osteosarcopenic obesity.

We recently conducted a retrospective analysis in over 250 postmenopausal women to identify those with OSO, osteopenic obesity, sarcopenic obesity, or obesity only (the latter having normal bone and muscle mass). First, women were classified as obese based on percent body fat [15]. We then evaluated and compared their functionalities, including handgrip strength, normal/brisk walking speed, and right/left leg stance [14]. Results showed that the OSO group presented with the lowest handgrip scores, the slowest normal and brisk walking speed, and the shortest time for each leg stance, although a statistically significant difference was reached only with the obese-only group. These findings indicate a greater tendency toward poorer functionality in women presenting with OSO compared to those with any other impaired condition (osteopenic obesity, sarcopenic obesity), but particularly to obese-only women, increasing the risk for bone fractures and immobility from the combined decline in bone and muscle mass, and infiltrated fat [14]. Therefore, the functional ability of individuals who present with multiple body composition impairments should not be neglected and could be used as an important additional (or even first) assessment in the diagnostic criteria for OSO.

At this point, it is not known whether the physical/physiological changes (e.g., decline in bone/muscle mass or certain hormones and rise in fat mass) precede the functional and strength decline (physical functionality and activities of daily living), or* vice versa*, or whether they all decline simultaneously although at varied rates [9]. The total number of fat cells an adult has may be determined in childhood [16]; however, it is the redistribution and expansion of adipocytes that occur with aging and other adverse conditions that leads to the negative health effects [17]. Fat serves no apparent functional benefit to locomotion other than being an energy storage. It may on the other hand impede [18] or indirectly inhibit function via adipokine induced low-grade chronic inflammation [4, 5].

Our objective is to introduce preliminary diagnostic criteria for OSO in older women based on two kinds of assessments: physical, via body composition measurements, and functional, via physical performance measures. The goal is that both groups of assessments could be easily performed and available in most clinical settings. These criteria are still preliminary due to limitations in diagnosing each of the conditions (particularly sarcopenia and sarcopenic obesity) within the spectrum of the final disorder, the OSO syndrome [19]. With more research and expanded measurements, the cut-off points may change and some adjustments may be necessary in the future. Likewise, the criteria for men need to be separately developed and outlined.

## 2. Proposed Identification and Diagnosis of Osteosarcopenic Obesity

### 2.1. Physical Assessment via Body Composition Measurements

The proposed diagnostic criteria for OSO in overweight/obese women (body fat ≥ 32%), based on the physical measurements, are presented in Table 1. These diagnostic criteria include measurements of bone, appendicular lean mass (predominantly muscle), and fat, the major components of the musculoskeletal system. These measurements can be performed in clinics with dual energy X-ray absorptiometry (DXA) technology in place. They include the diagnosis of the following.

#### 2.1.1. Osteopenia/Osteoporosis/Osteopenic Obesity

 Bone mineral density (BMD) assessed using *T*-scores ≤ −1.0 standard deviation (SD) of the femoral neck, proximal femur, or lumbar spine, based on the official diagnostic criteria for osteopenia/osteoporosis [20]: If body fat is ≥32% [15], the individual will also be classified into the osteopenic (or osteoporotic) obesity category.

#### 2.1.2. Sarcopenia/Sarcopenic Obesity

 Appendicular lean mass (ALM) measured by DXA and adjusted for both height (m) and fat mass (kg) [21] to diagnose sarcopenia: Negative residuals from a linear regression model are used to identify those individuals whose amount of ALM is lower than the predicted value for their height and fat mass, to diagnose sarcopenic obesity. The 20th percentile of the residual distribution is used as the cut-off point for sarcopenic obesity, with the equation: ALM = −17.4 + 18.3 × height (m) + 0.16 × body fat (kg). The cut-off was a residual value of −1.43 [14].

#### 2.1.3. Adiposity

 Fat mass ≥ 32% (obtained by DXA), as per newest recommendations for women by the American Society of Bariatric Physicians [15]: In that case, in addition to the diagnosis of both osteopenia/osteoporosis and sarcopenia, the osteosarcopenic obesity will be confirmed.

Therefore, the above physical assessment provides the diagnosis of the whole spectrum of conditions, including* osteopenia*,* sarcopenia, and/or obesity* as well as* osteopenic obesity*,* sarcopenic obesity*,* osteopenic sarcopenia, *and* osteosarcopenic obesity*, the last and most extreme stage in the spectrum (Table 1). Using DXA is practical because, as part of any planned bone density scans, body composition (lean and fat tissue) could be assessed as a part of the whole body scan at minimal additional cost.

### 2.2. Functional Assessments via Performance Measures

Based on the algorithm provided by the European Working Group on Sarcopenia in Older People (EWGSOP) [22], on the International Working Group on Sarcopenia [23], on the Foundation for the NIH Sarcopenia Project [24], and on the recently published motor assessment using the NIH Toolbox [25], as well as on our own studies in overweight/obese postmenopausal women [14], we propose the measurements of handgrip strength and the modified components of short physical performance battery (SPPB) to assess the overall physical performance for the categorization and the supplemental diagnosis of OSO. Handgrip strength is almost universally referred to as a measure of muscle strength in almost any assessment, as it correlates well with lower body strength [22] (which is harder to perform), as well as with overall muscle mass and BMD [26]. The SPPB original components included balance tests as side-by-side semi tandem and tandem stands; gait speed expressed in m/sec, performed at various speeds and lengths; and chair sit-to-stand, as a timed ability to rise from the chair [27]. Based on the subsequent modifications [15, 25, 28, 29] we propose the inclusion of the following tests under the SPPB:* one-leg stance* for balance, usual* gait speed* for endurance, and* sit-to-stand* chair test for lower extremity strength. Each test has its own cut-off values which could be incorporated into the total score for overall assessment of the functional performance. See Table 2.

#### 2.2.1. Handgrip Strength

(i) It is measured by the hand dynamometer, performed 2-3 times on each arm with the highest value taken. The participant extends the arm at 45°, holding the hand dynamometer, and on an exhale squeezes the hand dynamometer with maximum force. The clinician records the value in kg. The cut-off for sarcopenia and grip strength is ≤20 kg for women and ≤30 kg for men, based on data from the study in *n* = 1,030 participants, mostly women [22, 30].
(ii) Limitations for handgrip include the presence of rheumatoid arthritis, osteoarthritis, or other severe orthopedic and/or neurological disorders; this test might be uncomfortable for those participants and their scores appear to be too low [31, 32].

#### 2.2.2. One-Leg Stance

- For one-leg stance, the participant is asked to stand on one leg while lifting the contralateral limb, for up to 30 seconds, performed on both the right and left legs. The test stops when the participant touches any surface or lowers the contralateral limb to the ground or, ultimately, at the end of 30 seconds. This test is repeated twice with the highest value used for scoring [26]. An average score or cut-off for healthy older adults is 16 seconds, but younger participants will average 30 seconds or longer, further confirming a negative correlation of balance with aging [33, 34].
- Limitations for one-leg stance include participants with implants or arthritis in the hip or knee [35, 36].

#### 2.2.3. Gait Speed

- It is measured by timing the 4-meter (13.12 foot) usual walk. The 4-meter course is marked by two cones or pieces of tape. The participant starts at one end of the course, walking at her normal pace and walking past the other end of the course. The timing starts on the command “begin” and stops when one of the participant's feet is all the way across the 4-meter marker. Participants are allowed to use a cane or any other walking device they normally use when walking. The test is repeated twice, with the best time used for scoring. The cut-off value is ≤0.8 m/s, based on data from the study in 1,030 men and women of a wide-age range [22, 30, 37].
- Limitations for the walking test include mental impairment (assessed using the Mini Mental State Examination), history of chronic diseases such as stroke, congestive heart failure, Parkinson's Disease, active cancer, and neuropathy, inability to walk without crutches, and/or the presence of artificial limbs or prostheses [30, 37].

#### 2.2.4. Sit-to-Stand Chair Test

- The participant begins seated in an armless chair, arms crossed over her chest, back straight, and feet flat on the floor. She is then asked to rise from the chair and sit down again as many times as possible in a 30-second period. The number of consecutive chair sit-to-stand tests completed is recorded, with the last time the participant sat down in the chair being the final count. A “fit” older adult, without significant muscle loss, is defined as one who completes 20 (cut-off) or more sit-to-stands in a 30-second period [26, 38].
- Limitations for the sit-to-stand test include participants with severe orthopedic and neurological disorders, uncontrolled hypertension, and cardiovascular disease [32].

Table 2 outlines the assessment and scoring to determine the functional status of the participant. The score of “0” is assigned to each test performed barely at or below the given cut-off and the score of “1” to each test performed above the cut-off value. If there is an obvious limitation/disability for one of the tests (e.g., arthritis that prevents the handgrip or one-leg-stance), such test should not be performed and the score should be adjusted for the missing test. Based on the scores, four levels of functionality status could then be assigned:* major functional decline*,* moderate functional decline*,* minor functional decline,* and* no functional decline*, see Table 2. Therefore, the final diagnosis would consist of the* physical assessment* and* functional assessment*. For example, if a woman was diagnosed with sarcopenic obesity by the physical criteria and all functional scores were classified as a* major functional decline* (total score of 0 or 1), that woman would then be considered* sarcopenic obese with major functional decline*. Likewise, if the woman were diagnosed with OSO by the physical criteria but her functional assessment scores were 2-3 (at* minor functional decline*), this woman will be in a better health position, despite the OSO diagnosis, although the research shows that such situations are not likely to occur [14]. Within these parameters, any other condition can be assigned and quantified by the score for functionality.

## 3. Discussion and Conclusions

In view of the current debate as to which is a better prognostic measure for functionality, muscle mass, or muscle strength, we suggest having a dual diagnostic strategy to include age- and/or chronic disease-related physical and functional changes. This proposed model would ensure that both physical and functional changes are considered and would provide grounds for the subsequent and appropriate clinical or nutritional interventions. Within this proposed model, the classic definitions of osteoporosis, sarcopenia, and obesity are preserved to reflect the physical changes, and the functional changes would serve to include other practical components and ultimately help in directing better treatment options, within nutritional and physical activity domains. By including other components of the musculoskeletal system involved in locomotion, the gap in linking muscle mass, strength, and subsequent functionality may be reduced, and we may gain better insight when comparing functional and strength measures to changes in bone, muscle, and fat mass.

Briefly, we suggest two assessment steps for obtaining a more comprehensive diagnosis for OSO: (1) physical assessment as presented in Table 1. This could be performed in any clinical setting with the DXA technology. Thus the physical diagnosis would range from* osteopenia*,* sarcopenia, and/or obesity to osteopenic obesity*,* sarcopenic obesity*,* osteopenic sarcopenia, and osteosarcopenic obesity* (Table 1). Using DXA is practical because, as part of any planned bone density measurements, body composition (lean and fat tissue) could be assessed at minimal additional cost; (2) functional status assessment as presented in Table 2. Each of the measures could be easily performed in any clinical setting, as the tools are simple and portable and measurements are easily obtainable.

It is important to note that this proposed diagnostic model could be carried out by assessing functional status first and using the results to justify the body composition and bone density measurements. Either way, both the physical and functional changes could be used to better direct the treatment strategy. Because physical and functional changes require longitudinal measures, regular assessment of body composition, BMD, and functionality could be carried out starting in the 5th decade of life, as is currently recommended for the bone assessment in women.

In summary, osteopenia/osteoporosis has been classically regarded as bone loss with increased susceptibility to fractures and for a long time it was considered in isolation (without connecting to muscle or adipose tissue) [1]. However, in combination with excess fat, or with infiltration of fat into bone, the situation changes and could result in lower functionality and even higher fracture rates [1, 14]. Similarly, obesity and sarcopenia are classically defined as excess body fat and loss of muscle mass, respectively. However, new proposed definitions for sarcopenia and sarcopenic obesity focus more on muscle strength and functionality [22], which depend on both the infiltrated fat and the bone status [1]. Measuring functional changes may be appealing to the clinician, as they represent the real life scenario for people, and are simple enough to be performed at little or no cost in any healthcare facility. A remaining disconnect between muscle mass/size and strength/function may be explained by the fact that, in assessing strength and function, the entire musculoskeletal system is effectively involved: muscles, tendons, ligaments, bones, and cartilage [39], as well as the nervous system [40], and other functions including, blood flow, flexibility, core muscle strength, dynamic (postural) stability [41], and lung capacity. The combination of all these components further removes the measure of muscle mass itself from muscle strength and function, the latter two being indicators of other body functioning. Therefore, a combination of measures as proposed here can improve the assessment of body composition status and lead to better overall diagnosis of osteosarcopenic obesity and each of the conditions in a spectrum. Subsequent nutritional and/or physical activity measures could be incorporated as part of the standard care [2, 3].

## Figures and Tables

**Table 1:** Diagnostic criteria for osteosarcopenic obesity based on body composition (via dual energy X-ray absorptiometry, DXA).

Condition	*T*-score for BMD ≤−1.0 SD at the femoral neck, proximal femur, or lumbar spine	20th percentile of ALM for women	Fat mass ≥ 32% for women
Osteopenia/osteoporosis	Yes	No	No
Sarcopenia	No	Yes	No
Obesity	No	No	Yes
Osteopenic sarcopenia	Yes	Yes	No
Osteopenic obesity	Yes	No	Yes
Sarcopenic obesity	No	Yes	Yes

*Osteosarcopenic obesity*	*Yes*	*Yes*	*Yes*

BMD: bone mineral density; ALM: appendicular lean mass.

**Table 2:** Assessment and scoring of the functional performance and corresponding cut-off values.

Functional status	Handgrip strength (≤20 kg)	One-leg stance (≤16 sec)	Gait speed (≤0.8 m/sec)	Sit-to-stand chair test (≤20 times)	Total score
Major functional decline	0	0	0	0	**0**
Major functional decline^*∗*^	0	1	0	0	**1**
Moderate functional decline^*∗∗*^	0	0	1	1	**2**
Minor functional decline^*∗∗∗*^	0	1	1	1	**3**

No functional decline	1	**1**	1	1	4

The score of “0” is assigned to each test performed barely at or below the given cut-off and the score of “1” to each test performed above the cut-off value.

^**∗**^Any one performance could be scored as “1,” if it is above the cut-off for a given functionality.

^**∗∗**^Any two performances could be scored as “1,” if they are above the cut-off for given functionality.

^**∗∗∗**^Any three performances could be scored as “1,” if they are above the cut-off for given functionality.

A total score of 0 or 1 indicates a state of *major functional decline.*

A total score of 2 indicates *moderate functional decline*.

A total score of 3 indicates *minor functional decline*.

A total score of 4 indicates *no functional decline.*
